# The flavohaemoprotein hmp maintains redox homeostasis in response to reactive oxygen and nitrogen species in *Corynebacterium glutamicum*

**DOI:** 10.1186/s12934-023-02160-9

**Published:** 2023-08-18

**Authors:** Ziqin Jiang, Jingyi Guan, Tingting Liu, Chunyu Shangguan, Meijuan Xu, Zhiming Rao

**Affiliations:** 1 The Key Laboratory of Industrial Biotechnology, School of Biotechnology, Ministry of Education, Jiangnan University, 1800 Lihu Road, Wuxi, Jiangsu 214122 China; 2 Yantai Shinho Enterprise Foods Co., Ltd, Yantai, 265503 China

**Keywords:** Flavohaemoprotein, *Corynebacterium glutamicum*, Redox homeostasis, L-arginine

## Abstract

**Background:**

During the production of L-arginine through high dissolved oxygen and nitrogen supply fermentation, the industrial workhorse *Corynebacterium glutamicum* is exposed to oxidative stress. This generates reactive oxygen species (ROS) and reactive nitrogen species (RNS), which are harmful to the bacteria. To address the issue and to maintain redox homeostasis during fermentation, the flavohaemoprotein (Hmp) was employed.

**Results:**

The results showed that the overexpression of Hmp led to a decrease in ROS and RNS content by 9.4% and 22.7%, respectively, and improved the survivability of strains. When the strains were treated with H_2_O_2_ and NaNO_2_, the RT-qPCR analysis indicated an up-regulation of ammonium absorption and transporter genes *amtB* and *glnD*. Conversely, the deletion of *hmp* gives rise to the up-regulation of eight oxidative stress-related genes. These findings suggested that *hmp* is associated with oxidative stress and intracellular nitrogen metabolism genes. Finally, we released the inhibitory effect of ArnR on *hmp*. The Cc-ΔarnR-hmp strain produced 48.4 g/L L-arginine during batch-feeding fermentation, 34.3% higher than the original strain.

**Conclusions:**

This report revealed the influence of dissolved oxygen and nitrogen concentration on reactive species of *Corynebacterium glutamicum* and the role of the Hmp in coping with oxidative stress. The Hmp first demonstrates related to redox homeostasis and nitrite metabolism, providing a feasible strategy for improving the robustness of strains.

**Supplementary Information:**

The online version contains supplementary material available at 10.1186/s12934-023-02160-9.

## Background

Bacteria in industrial fermentation environments are frequently suffer from a range of artificially driven stresses, such as temperature, pH, osmotic, nutrient starvation, and oxidation [1, 2]. *Corynebacterium glutamicum* is a widely used industrial strain for producing critical amino acids and organic acids under high dissolved oxygen and nitrogen sources. During the fermentation process, unfavorable conditions can lead to oxidative stress, including reactive oxygen (ROS) and reactive nitrogen (RNS) [3, 4]. The ROS and RNS, like hydroxyl radical (OH^−^), superoxide anion (O2^−^), nitric oxide radical (NO^−^), and peroxynitrite (ONOO^−^), which caused damage to bacteria such as mutation, metabolic pathway disruption, and growth inhibition [5–7]. Under aerobic conditions, bacteria need to accumulate biomass and product quickly to have high carbon flux to ferment [8]. However, aerobic metabolism comes with the generation of reactive species that damage biological macromolecules, including nucleic acids, lipids, proteins, and carbohydrates [9]. This damage is detrimental to the growth patterns of various organisms [10].

Previous research has indicated that bacteria have developed various strategies to cope with redox stresses [11], including non-enzymatic and enzymatic antioxidant systems [12]. Mycothiol is the major non-enzymatic antioxidant in high-GC Gram-positive bacteria, which can maintain intracellular redox homeostasis by oxidizing into its disulfide form mycothiol disulfide under oxidative conditions [13–18]. Highly efficient enzymatic antioxidants include superoxide dismutase SOD, catalase CAT, methionine sulfoxide reductases (Msr), peroxiredoxins mycothiol peroxidase (Mpx), and thiol-peroxidase (Tpx) [19–24]. SOD and Mpx are the main antioxidant enzymes responsible for maintaining the redox balance of aerobic microorganisms. Research has shown that the generation rates of free radicals are proportional to the levels of redox enzymes [25, 26]. Moreover, transcriptional regulators respond to different types of ROS and induce the expression of related enzyme genes to eliminate ROS [27–32].

The Hmp plays a part in the redox process of microorganisms [33], protecting cells from oxidative pressure. It is composed of a hemoglobin-like domain with a β-type heme at the N-terminus and a ferredoxin NADP^+^ reductase domain with FAD and NADPH binding domain at the C-terminus [34]. Hmp has two functions, acting as an oxygen-dependent NO dioxygenase (NOD) that converts NO^−^ and O_2_ into inert nitrates and catalyzes regeneration through a flavin-dependent reduction reaction [35], and as an oxygen-independent NO reductase (NOR) that converts NO^−^ into non-toxic nitrous oxide [36]. The NO-sensing regulator ArnR senses NO- through metal centers and nitrosylated cysteine residues, inhibiting the expression of the nitrate reductase operon *narKGHJI* and *hmp* under aerobic conditions, thereby avoiding anaerobic nitrate respiration [35, 37].

**Fig. 1:**
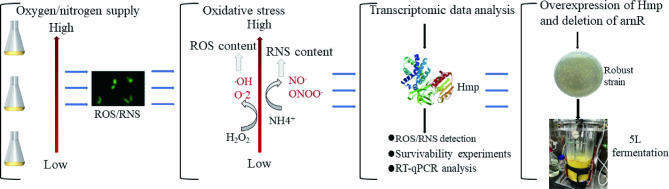
The role of Hmp in maintaining cellular redox state and its impact on L-arginine fermentation. Exploring the process of the role of Hmp in maintaining the intracellular redox state in response to ROS and RNS during L-arginine fermentation under high oxygen and nitrogen supply conditions in Cc5-5, and investigating the impact of the recombinant strain Cc-ΔarnR-hmp on L-arginine fermentation

L-arginine has the highest N:C ratio, the high oxygen and nitrogen supply essential to L-arginine fermentation conditions [33, 38]. Our previous study obtained an high-yielding L-arginine strain Cc5-5 by screening and mutation breeding (*Corynebacterium glutamicum* var. CCTCC AB 2,021,051, Cc5-5) [39]. This study investigated the formation of ROS/RNS during L-arginine fermentation under high oxygen and nitrogen supply conditions in Cc5-5, and furthermore, it explored the role of Hmp in maintaining the intracellular redox state and the inhibitory effect of ArnR on hmp (Fig. 1).

## Results

### Accumulation of ROS and RNS with the increasing oxygen and nitrogen supply

**Fig. 2:**
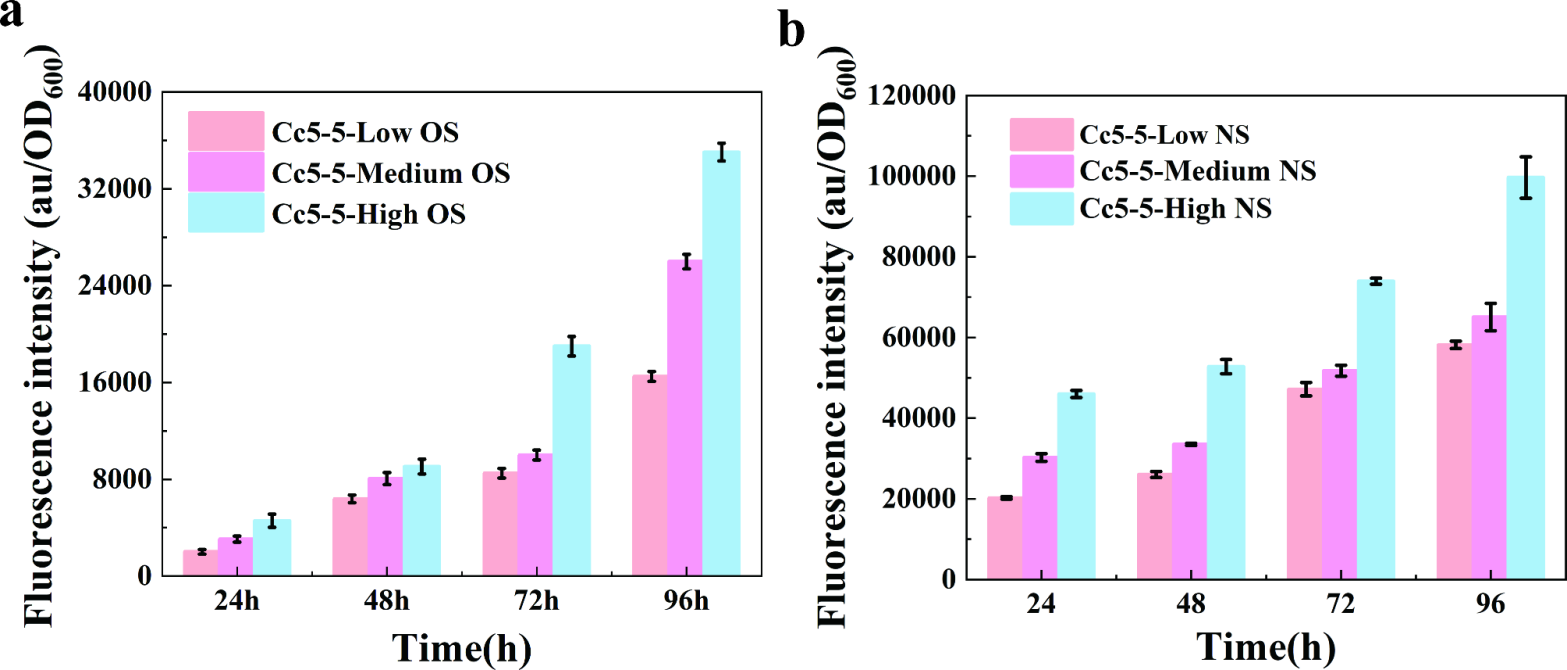
Increasing the supply of oxygen and nitrogen leads to an accumulation of ROS and RNS **a.** Determination of the intracellular ROS content of Cc5-5 was determined at different times and under different oxygen supply conditions by the DCFH-DA fluorescent probe. **b**. Determination of the intracellular RNS content of Cc5-5 at different times and under different nitrogen supply conditions by theO52 fluorescent probe

In this study, we aimed to investigate the impact of oxygen and nitrogen supply on the formation of intracellular ROS and RNS during L-arginine fermentation by Cc5-5. To achieve this, cells were cultured under different oxygen and nitrogen concentration and used corresponding fluorescent probes to determine intracellular ROS and RNS production during fermentation at 24 h, 48 h, 72 h, and 96 h. Results showed that oxygen supply had an effect on aerobic respiration and metabolic intensity, and the high oxygen supply increased intracellular ROS production (Fig. 2a). Although the intracellular ROS content of Cc5-5 remained relatively stable during the early stage of fermentation, but significantly increased during the late stages of fermentation under high oxygen supply conditions. For instance, at 96 h, ROS production increased by nearly 9 times compared to 24 h, and was 2.1 and 1.3 times higher than medium and low oxygen supply, respectively. As expected, the oxygen supply is positively correlated with the generation rate of intracellular ROS. The stepwise increase of nitrogen supplies was found to increase RNS production (Fig. 2b), and at 96 h, the Cc5-5 fermentation under high nitrogen supply had significantly higher RNS production than other nitrogen supply and fermentation stages. The laser confocal microscope was used to observe the changes in fluorescent and cell morphology (Fig. S1). Our previous studies followed the results [40], indicating that the Cc5-5 experiences oxidative stress during the fermentation of high oxygen and nitrogen supply because of the accumulation of endogenous ROS and RNS.

### Overexpression of Hmp enhanced resistance to ROS and RNS

**Fig. 3:**
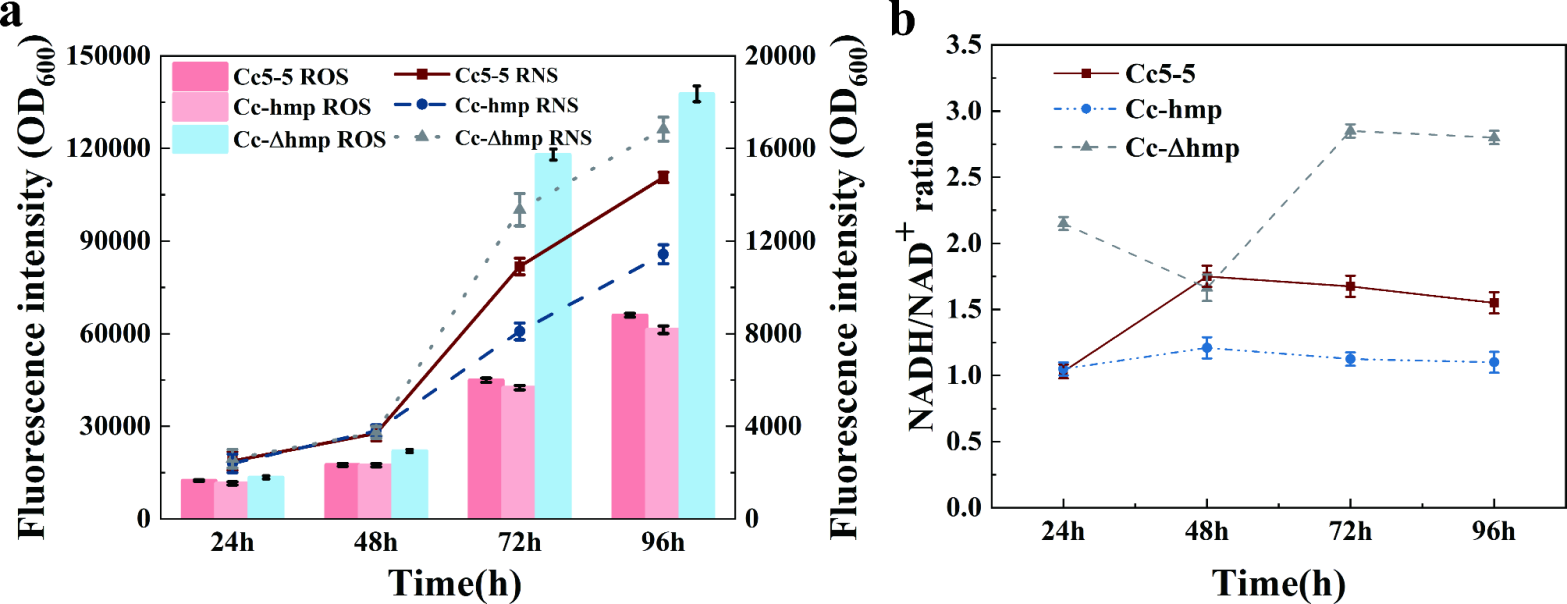
Hmp enhanced resistance to reactive species and utilized NADH as the cofactor **(a)** Determination of the intracellular RNS and ROS content of Cc5-5, Cc-hmp and Cc-Δhmp strains at different times under high oxygen and nitrogen supply condition by the O52 and DCFH-DA fluorescent probe. **(b)** Determination of the intracellular NADH/NAD^+^ ratio of Cc5-5, Cc-hmp and Cc-Δhmp strains under high oxygen and nitrogen supply condition at different times

As inevitable products in an environment rich in oxygen and nitrogen, ROS and RNS can damage cells and disrupt redox homeostasis. The previous transcriptome analysis under high oxygen and nitrogen supply fermentation conditions found an increase in the transcription level of the *hmp* gene. Therefore, we speculate that the *hmp* gene was responsible for the oxidative stress induced by high oxygen and nitrogen supply fermentation conditions. To verify this hypothesis, we constructed an hmp overexpression strain Cc-hmp and hmp deletion strain Cc-Δhmp and then determined the content of ROS and RNS during high oxygen and nitrogen supply fermentation. Since the oxidation stress is low at the initial stage of fermentation, the content of ROS and RNS in Cc-hmp, Cc-Δhmp and Cc5-5 was not notably different. However, after 96 h of fermentation, the intracellular ROS content of Cc-hmp was 9.4% lower than Cc5-5 (Pvalue < 0.05), while Cc-Δhmp was 2.1 times higher than Cc5-5. The intracellular RNS of Cc-hmp decreased by 22.7%, whereas that of Cc-Δhmp increased by 20.9% at 96 h (Fig. 3a). The results indicated that deleting the hmp gene reduced the intracellular antioxidant capacity. In contrast, overexpression of the *hmp* gene improved oxidation resistance. Hmp protects cells from harmful intracellular ROS and RNS in the later stage of fermentation, which is beneficial to maintain intracellular redox homeostasis.

### NADH as the cofactor of hmp to maintain redox homeostasis

Previous studies have shown that Hmp used NADH or NADPH as cofactors to protect cells from oxidative damage during stress conditions [41]. To investigate the type of cofactors utilized by Cc5-5 Hmp, the Hmp was purified to determine the enzyme activity of NAD(P)H oxidase. The Hmp activities of Cc-hmp, Cc-Δhmp and Cc5-5 strains were determined during fermentation. The specific enzyme activity of Cc-hmp, with NADH as a substrate, showed a 30.5% increase compared with Cc5-5, while the specific enzyme activity of Cc-Δhmp demonstrated a 12.7% decrease (Pvalue < 0.05). These results suggested that Hmp uses NADH as a cofactor to cope with oxidative stress (Table 1).

**Table 1:** NADH(P) oxidase activity of Hmp protein in Cc5-5 and recombinant strains(N = 3)

Strains	Substrate	Flavin	Specific enzyme activity(mU·mg^− 1^ )
Cc5-5	NADH	FAD	121.6 ± 5.1
	NADPH	FAD	4.2 ± 0.21
Cc-hmp	NADH	FAD	158.7 ± 7.4
	NADPH	FAD	5.2 ± 0.3
Cc-Δhmp	NADH	FAD	106.2 ± 6.4
	NADPH	FAD	3.4 ± 0.3

To further confirm the results, we measured the intracellular NADH/NAD^+^ ratio of the recombinant strain under high dissolved oxygen fermentation conditions. At 24 h, the intracellular NADH/NAD^+^ of Cc-Δhmp was found to be 1.9 times higher than that of Cc5-5, while the NADH/NAD^+^ in Cc-hmp did not show a significant difference with Cc5-5. With the extension of time, from 48 to 96 h, the NADH/NAD^+^ in the cell of Cc5-5 and Cc-hmp showed a slow downward trend. In contrast, as shown in Fig. 3b the NADH/NAD^+^ in Cc-Δhmp cells was 1.7 times higher than that of Cc5-5 at 96 h, while Cc-hmp showed a 34.1% decrease (Pvalue < 0.05). These results were consistent with with the fact that NADH is the cofactor of Hmp.

### Effect of hmp on the survivability of Cc5-5 under oxidative stress

The purpose of the survival experiments was to assess the role of Hmp in mitigating stress induced by ROS and RNS. The survival rates of Cc-hmp and Cc-Δhmp were compared when treated with different concentrations of H_2_O_2_ and NaNO_2_. The results showed (Table 2) that when treated with 50 mmol/L H_2_O_2_, the survival rate of Cc-hmp was equivalent to Cc5-5. However, when the concentration rate of H_2_O_2,_ was increased to 100 mmol/L H_2_O_2,_ the survival rate of Cc-Δhmp decreased by 16% compared with Cc5-5 while the survival rate of Cc-hmp increased by 14% compared to Cc5-5. When 50 mmol/L NaNO_2_ was added, the survival rate of Cc-Δhmp decreased by 21% compared with Cc5-5. As expected, the survival rate of Cc-hmp improved by approximately 10% compared to Cc5-5. With the concentration of NaNO_2_ increased to 100 mmol/L, the survival rate of Cc-Δhmp decreased by 31% compared with Cc5-5, while the survival rate of Cc-hmp increased by 12% (Pvalue < 0.05). The results indicated that overexpression of the Hmp improved the survivability of the Cc5-5 strain under oxidation treatment.

**Table 2:** Survivability of Cc5-5 and recombinant strains(N = 3)

Strains	H2O2 (mM)	Survival rate (%)	Na2O2 (mM)	Survival rate(%)
Cc5-5	50	77.8 ± 1.8	50	76.0 ± 2.8
	100	50.5 ± 2.3	100	54.4 ± 3.1
Cc-hmp	50	80.6 ± 1.7	50	86.1 ± 1.5
	100	71.5 ± 3.2	100	67.5 ± 2.9
Cc-Δhmp	50	74.2 ± 2.6	50	55.2 ± 3.6
	100	37.8 ± 1.6	100	22.6 ± 3.0

### Upregulation of hmp and oxidative stress-related genes under oxidative stress

To detect the change in *hmp* gene expression level under oxidation treatment, RT-qPCR experiments were conducted. The results showed a slight increase in the expression level of the *hmp* gene when treated with 50 mmol/L and 100 mmol/L H_2_O_2_. Exposure to 50 mmol/L and 100 mmol/L NaNO_2_ increased the hmp gene expression level by 2.6 and 4.6-fold, respectively (Fig. S2). These results indicated that oxidative stress increased the expression level of hmp.

**Fig. 4:**
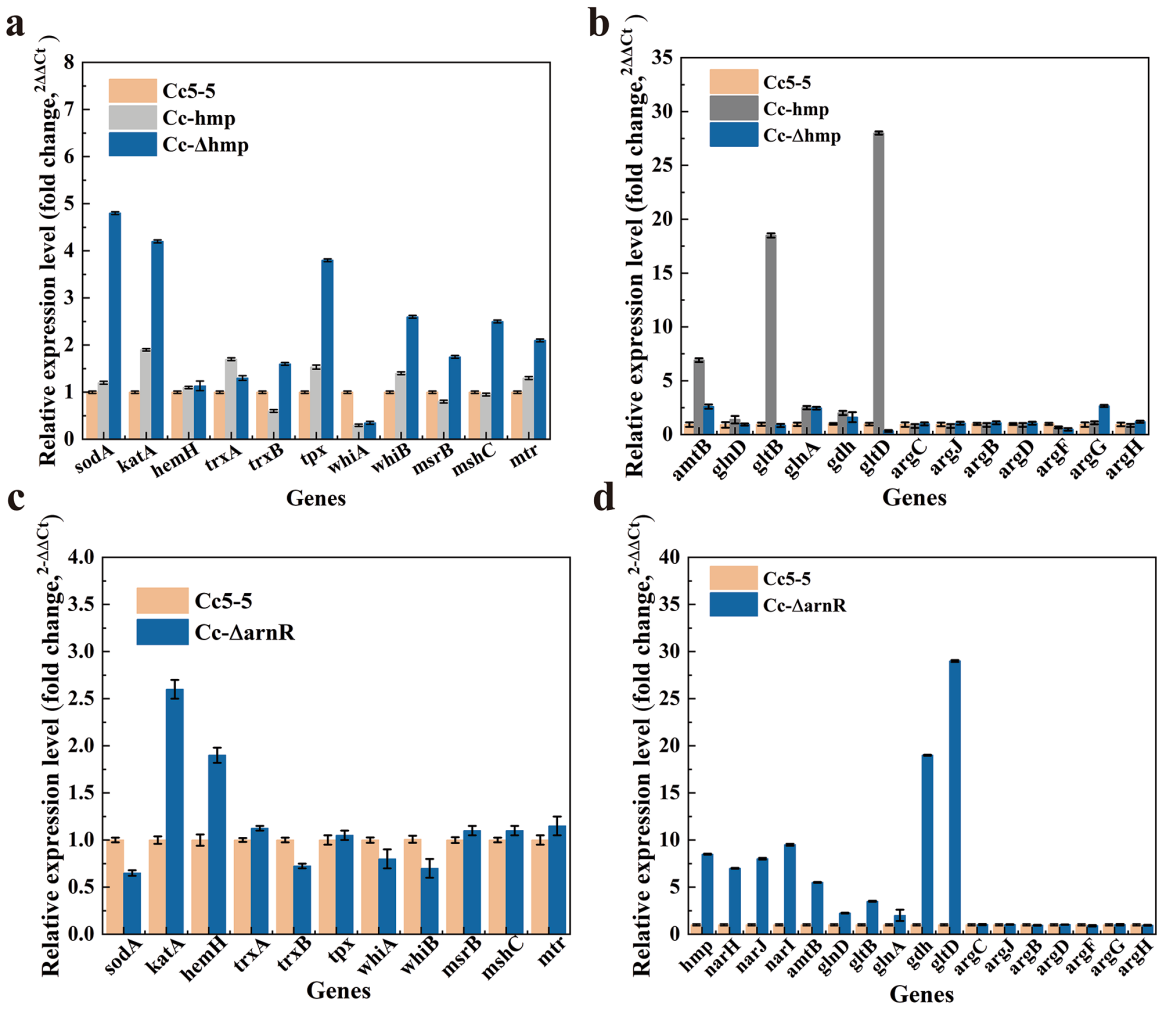
RT-qPCR analysis of *hmp* and arnR impact on the transcription levels of key genes in oxidative stress and nitrogen metabolism. **a.** the *hmp* impact on oxidative stress-related genes. **b**.the *hmp* impact on nitrogen metabolism genes. **c**. the *arnR* impact on oxidative stress-related genes. **d**. the arnR impact on nitrogen metabolism genes. The standard deviations of the data points were obtained from triplicate measurements and denoted by error bars

To gain a deeper understanding of how Hmp affects intracellular redox homeostasis, we conducted further analysis of the expression levels of oxidative stress, nitrogen metabolism and L-arginine biosynthesis genes in Cc5-5, Cc-hmp and Cc- Δhmp by RT-qPCR. As shown in Fig. 4a, the expression levels of *sodA* (superoxide dismutase), *katA* (catalase), *tpx* (thiol peroxidase), *whiB* (transcriptional regulator WhiB), *msrB* (Peptide-methionine -S-oxide reductase), *mshC* (1D-myoinositol 2-amino-2-deoxy-alpha-D-glucopyranoside ligase), *mtr* (mycothione reductase) and *trxB* (thioredoxin-disulfide reductase) were up-regulated in Cc-Δhmp. These results indicated that deletion of hmp resulted in decreased intracellular antioxidant capacity and up-regulated expression levels of oxidative stress-related genes to cope with ROS and RNS. As shown in Fig. 4b, the expression levels involved in ammonium absorption-related genes *gltB* (large subunit of glutamate synthase) and *gltD* (small subunit of glutamate synthase) were most significantly up-regulated in Cc-*hmp*, showing18.3- and 27.5-fold increases compared to Cc5-5, respectively. The ammonium transporter AmtB and adenylyltransferase GlnD encoding genes *amtB* and *glnD* were up-regulated 5.6 times and 1.5 times, respectively. Still, no significant differences were found in the expression levels of genes related to the L-arginine synthesis gene cluster. The up-regulation of these genes will affect the nitrogen metabolism pathway, promote the utilization of nitrogen sources, reduce the formation of intracellular RNS, and further affect the synthesis of L-arginine.

### Releasing the inhibition of ArnR on Hmp enhances cellular redox homeostasis

ArnR is a nitrogen-related regulatory factor that inhibits the expression of *hmp* under aerobic conditions, which is unfavorable for the function of Hmp [42]. To confirm if ArnR inhibited *hmp* expression under aerobic conditions, we constructed a strain Cc-ΔarnR deficient in *arnR*. The expression level of *hmp* in Cc-ΔarnR was found to be 8.1 times higher than in Cc5-5 (Fig. S3). The deletion of *arnR* relieved the inhibitory effect of transcription regulator ArnR on *hmp*, thus improving the expression level of Hmp. Subsequently, we used RTq-PCR to investigate the expression levels of oxidative stress-related genes in the the Cc5-5 and Cc-ΔarnR strains. There was no significant difference in the antioxidant capacity of the cell after deleting *arnR*. However, the RTq-PCR analysis revealed that mRNA levels of oxidative stress-related genes such as *sodA*, *tpx*, *whiB*, *msrB*, *mshC*, *mtr* and *trxB* did not change significantly. Only the expression levels of *katA and hemH* were up-regulated by 2.5- and 1.8-fold, respectively (Fig. 4c).

On the other hand, we studied the relationship between ArnR and nitrogen metabolism. The deletion of *arnR* increased the expression levels of genes regulated by ArnR, such as *hmp*, *narH*, *narJ*, and *narI*, which were up-regulated by 8.1-, 7.0-, 7.9-, and 9.8-fold, respectively. Genes associated with ammonium absorption, such as *gltB* and *gltD*, showed a remarkable upregulation of 18.6 and 29.3-fold, respectively, while the expression levels of an ammonium transporter and adenylate transferase genes *amtB* and *glnD* were up-regulated 5.1 and 1.6-fold, respectively (Fig. 4d). The results of RT-qPCR experiments showed that deletion of *arnR* enhanced the nitrogen transport and absorption capacity. Releasing the inhibition of ArnR on the Hmp strengthens the ability of nitrogen transport, which leads to the rapid utilization of nitrogen sources, reduces the formation of RNS, and consequently enhances the resistance of cells to reactive nitrogen.

### Effects of recombinant strains on the production of L-arginine fermentation

In previous experiments, it was indicated that Hmp maintained cell homeostasis by reducing the formation of ROS and RNS. Therefore, to verify whether the Hmp boost L-arginine production by improving cell redox homeostasis, a 5 L batch fermentation of Cc5-5, Cc-hmp, Cc-ΔarnR strains was conducted. The dry cell weight, glucose concentration and L-arginine production during the fermentation process were measured.

**Fig. 5:**
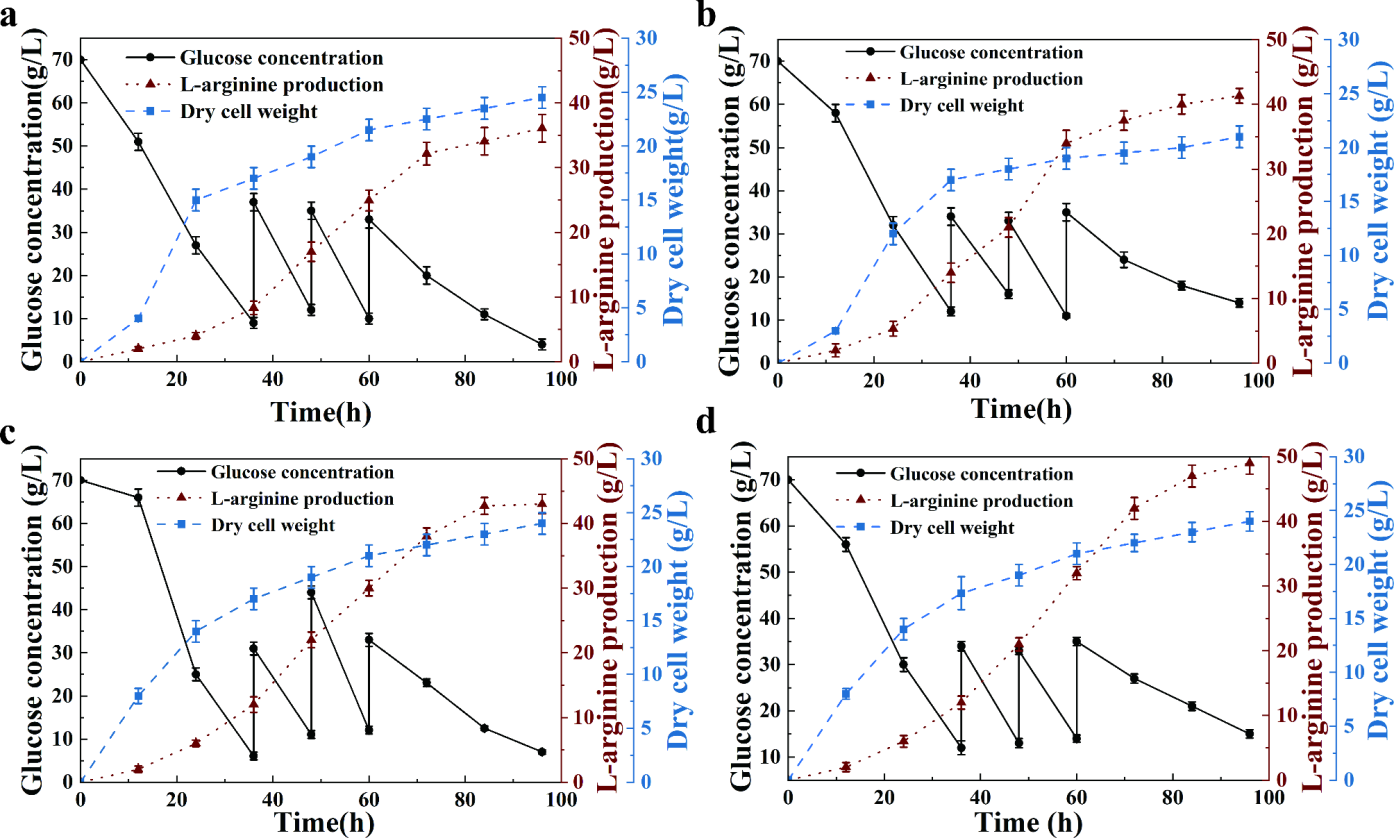
Time course of L-Arginine fermentations of Cc 5–5 and recombinant strains in 5-L fermenters. **(a)** Fermentation-related curve of Cc5-5. **(b)** Fermentation-related curve of Cc-hmp. **(c)** Fermentation-related curve of Cc-ΔarnR. **(d)** Fermentation-related curve of Cc-ΔarnR-hmp. Experiments were conducted in triplicate. Error bars indicate ± SD

Cc-hmp had a slightly slower glucose consumption rate than Cc5-5 in the later stage of the fermentation process at 60 h (Fig. 5a). However, Cc-hmp produced 43.2 ± 2.4 g/L L- arginine with a productivity of 21.4 ± 1.2 mg/g dry cell weight/h, 19.7% and 38.9% higher than Cc5-5 (Fig. 5b). The increase in L-arginine production was attributed to the effective removal of harmful reactive species during the fermentation process by Hmp. Deletion of *arnR* significantly improved the hmp expression level. The survivability of Cc-ΔarnR did not show a significant increase, and produced 42.7 ± 2.1 g/L L- arginine with a productivity of 18.6 ± 1.8 mg/g dry cell weight /h, respectively (Fig. 5c). These values increased by 18.3% and 20.8%, respectively, compared to Cc5-5. Releasing the transcriptional inhibition of ArnR on Hmp will enhance the transport capacity of ammonium, reduce the formation of RNS and ROS, and further increase the production of L-arginine.

To obtain an engineered strain with high production of L-arginine, the Cc-ΔarnR-hmp strain was constructed. Compared with Cc5-5, Cc-ΔarnR-hmp showed slightly slower glucose consumption at 60 h, but no significant differences in growth. The Cc-ΔarnR-hmp produced 48.4 ± 2.0 g/L L-arginine with a productivity of 23.4 ± 1.6 mg/g dry cell weight /h, 34.3% and 48.8% higher than that of Cc5-5 (Fig. 5d). Our findings suggest that Hmp could improve the intracellular environment during the fermentation process and then increase the yield of target metabolites. Hmp is crucial for bacteria growth and L-arginine anabolism.

## Discussion

In aerobic conditions, bacteria have high carbon flux to ferment, enabling them to rapidly accumulate biomass and production [43]. However, aerobic metabolism is accompanied by the generation of reactive species, which are detrimental to the growth patterns of various organisms [44]. *Corynebacterium glutamicum* is an industrial production strain that produces many critical amino acids and serves as an ideal microbial chassis cells for constructing metabolic networks [45]. The production of L-arginine requires a high level of dissolved oxygen and high nitrogen source during fermentation, which can cause cells to suffer from oxidative and nitrosation stress [38, 45]. Through our research we confirmed that a large number of reactive species would accumulate during the middle and late fermentation under high dissolved oxygen and nitrogen source fermentation, which poses a huge threat to cell growth and metabolism.

Many aerobic microorganisms need an enzymatic antioxidant to maintain the redox balance of bacterial cells [46]. The Hmp protein is involved in the redox process of microorganisms and has a stress-protective effect on nitrite under anaerobic and micro-aerobic conditions. In this work, we demonstrated that Hmp plays a role in maintaining redox homeostasis by protecting cells from RNS and ROS and uses NADH as a cofactor in the late-stage of high dissolved oxygen fermentation. The deletion of *hmp* significantly affected the expression of oxidative stress-related genes such as *sodA and katA*. Furthermore, Hmp is related to intracellular nitrogen metabolism as well. The RT-qPCR experiments showed that the expression levels genes related to ammonium absorption and transporter were up-regulated, such as *gltB* and *gltD*,* amtB* and *glnD*. These results revealed that Hmp positively regulated nitrogen metabolism transcription levels and promoted nitrogen absorption and transport.

Since the ArnR inhibits the expression of *narKGHJI* nitrate reductase operon and *hmp* [42], the deletion of ArnR released the repression of Hmp protein and promoted the utilization of nitrogen sources. In fed-batch fermentation, we found that removing the inhibitory effect of ArnR on Hmp created an increase in L-arginine production by Cc-ΔarnR-hmp, which produced 48.4 ± 2.0 L-arginine, a 34.3% improvement over the original strain. These results strongly support the idea that Hmp plays a vital role in enhancing cellular redox homeostasis.

## Conclusion

This work indicated when cells are exposed to high oxygen and nitrogen supply in L-arginine fermentation, Hmp, with the NADH as a cofactor, helps to scavenge ROS and RNS to maintain redox homeostasis and protect cells from the determination of oxidative stress. Overexpression of Hmp led to decreased reactive species and improved survivability. When treated with H_2_O_2_ and NaNO_2,_ the hmp and oxidative stress-related genes were up-regulated, as shown by RT-qPCR experiments. Hmp is related to genes involved in intracellular nitrogen metabolisms, suggesting that Hmp promoted the nitrogen absorption and transport. The expression of the hmp gene is repressed by ArnR, but deletion of arnR failed to show a significant growth defect under aerobic and anaerobic conditions [35]. However, the deletion of ArnR released the repression of Hmp protein and promoted the utilization of nitrogen sources. The recombinant strain Cc-hmp-ΔarnR shows a significant impact on L-arginine production under fermentation. Thus, overexpression of Hmp is a promising strategy for developing highly robust industrial production strains.

## Materials and methods

### Bacterial strains and cultivation conditions

All bacterial strains and plasmids used in this study are listed in Table S1. *Escherichia coli* strains JM109 and BL21 (DE3) were used as hosts for plasmid construction and heterologous expression, respectively. *Escherichia coli is* cultured at 37 °C and 180 rpm for 12 h in LB medium. *Corynebacterium glutamicum* strains were cultured at 30 °C for 18 h in LBG medium (LB medium supplemented with 5 g/L glucose). The minimal medium CGXII (supplemented with 40 g L^− 1^glucose) was used to culture *Corynebacterium glutamicum* for survival experiments. Seed medium (g·L^− 1^): Yeast extract 20, Glucose 50, (NH4)_2_SO_4_ 20, KH_2_PO_4_ 1, MgSO_4_·7H_2_O 0.5, pH 7.2. Fed-batch fermentation medium (g·L^− 1^) composed of Yeast extract 20, Glucose 70, (NH4)_2_SO_4_ 40, MgSO_4_·7H_2_O 0.5, MnSO_4_ 0.02, KH_2_PO_4_ 1.5, FeSO_4_·7H_2_O 0.02, pH 7.2. For the L-arginine fermenter culture, the seed medium (150 mL) was transferred to 5 L fermenters (BIOTECH-5BG, Baoxing Co, Shanghai, China) containing 2.5 L of fermentation medium. The fermentation experiments were performed at 30 °C, and the pH was maintained at 7.0 by automatically adding 50% NH_3_·H_2_O solution [47]. The dissolved oxygen level is controlled by the speed of the reciprocating shaker. Low dissolved oxygen (Low-DO): 110r·min^− 1^, medium dissolved oxygen 165r·min^− 1^(Medium-DO), high dissolved oxygen 220r·min^− 1^(High-DO).

### Construction of the recombinant strains

The primers used in the construction of the recombinant strains are shown in Table S2. The gene coding for hmp was amplified by PCR using Cc5-5 genomic DNA as the template. The amplified fragment and empty expression vector plasmid pXMJ19 were digested by restriction enzymes *EcoR* I and *Hind* III. The fragment was further ligated with a similarly digested pXMJ19 plasmid, obtaining pXMJ19-hmp. The constructed vector was transformed into *E. coli* BL21(DE3) competent cells and cultured in LB medium (containing chloramphenicol 50 mg/mL) for 12 h. The protein expression in Cc5-5 was induced by adding 0.5 mM isopropyl β -D-1-thiogalactopyranoside (IPTG) and analyzed by SDS-PAGE to verify the successful construction of the Hmp overexpression strain Cc-hmp.

The deletion mutants were achieved by a two-step homologous recombination method based on the plasmid pK18*mobsacB*. The Cc5-5 genomic DNA was extracted as a template for PCR amplification to obtain the upstream and downstream regions of the *hmp* and *arnR* genes. In the next step, the upstream and downstream PCR fragments were fused by overlap extension polymerase chain reaction (OE-PCR) with the primer pairs pK18-ΔHmp-1, pK18-ΔHmp-4, pK18-ΔarnR-1 and pK18-ΔarnR-4, respectively. Then the resulting DNA fragments were ligated with the suicide plasmid pK18*mobsacB* and transformed into the competent *E. coli* JM109 to create the pK18*mobsacB*-Δhmp and pK18*mobsacB*-ΔarnR plasmid. The plasmids were transformed into Cc5-5 by the electroporation method. The Δhmp and ΔarnR mutant was obtained through a two-step selection strategy and confirmed by colony-PCR. The first was selected in kanamycin-containing mediums and then selected hmp, arnR deletion mutant strains with LBGS medium (peptone 10 g/L, yeast extract 5 g/L, sodium chloride 10 g/L, glucose 10 g/L, sucrose 100 g/L, pH 7.0). The genomes of the strains that successfully knocked out *hmp* and *arnR* genes were extracted and sequenced to confirm.

### Detection of intracellular reactive oxygen species

ROS levels were investigated by the fluorescent probe 2′,7′-dichlorodihydro fluorescein diacetate (DCFH-DA) (Nanjing Jiancheng Research Institute). The fluorescence intensity was detected at the excitation wavelength of 502 nm, and emission wavelength of 530 nm. All tests were performed in triplicate.

### Detection of intracellular reactive nitrogen level

RNS levers were detected using the bacterial reactive nitrogen detection fluorescent probe O52 of Shanghai Beibo Biotechnology. The fluorescence intensity was detected at the excitation wavelength of 488 nm and emission wavelength of 526 nm. All tests were performed in triplicate.

### Determination of SOD and CAT enzyme activity

SOD enzyme activity was measured by SOD kit (Beyotime, Shanghai, China). CAT enzyme activity was detected by the kit of Nanjing Jiancheng Institute. All tests were performed in triplicate.

### Determination of the NADP^+^, NADPH and NADP^+^/NADPH value

The NADP^+^ and NADPH were detected by NADP^+^/NADPH Assay Kit with WST-8(Beyotime, Shanghai, China). All tests were performed in triplicate.

### Survival experiments

The Cc5-5, Cc-hmp, and Cc-Δhmp strains were cultivated in CGXII medium for 48 h, which grew to the exponential phase. The OD_600_ was adjusted to the same value, then H_2_O_2_ and NaNO_2_ were added to further cultured cells for 3 h. Adjust 1mL sample to the appropriate concentration and spread the fresh LBG plate. The colonies were counted after 2 ~ 3 days of incubation at 32 °C. The survival rates were calculated as the number of colony-forming units (CFUs).

### RT-qPCR analysis

Bacterial total RNA was extracted by Tiangen Bio’s RNAprep PureCell/Bacteria Kit (DP430) according to the manufacturer’s protocol. Reverse transcription was performed by Novozymes’ HiScript® II Q RT SuperMix for qPCR (+ gDNA wiper) (R323-01) kit. Sample preparation was performed by the ChamQTM Universal SYBR® qPCR Master MixCQ711StepOnePlu. RT-qPCR analysis was performed by fluorescence quantitative PCR instrument (Applied Biosystems), and the 16sRNA gene was selected as an internal reference gene for quantification. The experiment was repeated three times, and the average threshold cycle was calculated. Finally, the relative gene expression levels were calculated according to the 2^–∆∆Ct^ method. The RT-qPCR primers used in this study are shown in Table S3.

### Determination of flavohaemoprotein hmp enzyme activity

The enzyme activity was analyzed by measuring the change of absorbance at 340 nm to monitor the consumption of NAD(P)H. The reaction system contains 0.1 mol·L^− 1^ Tris-HCl (pH 7.5), 100 µmol·L^− 1^ NAD(P)H, 1 µmol·L^− 1^ oxidized flavin adenine dinucleotide (FAD) and A certain amount of enzyme solution. Enzyme activity is defined as at 30 °C and pH 7.5 reaction conditions, the amount of enzyme catalyzed the 1 µmol·L^− 1^ NAD(P)H per minute is one enzyme activity unit (1 U).

### Fed-batch fermentation

Analytical methods: Cell concentration was monitored by measuring OD_600_ using a spectrophotometer (UNICOTM-UV2000; Shanghai, China) after dissolving CaCO_3_ with 0.125 M HCl, and dry cell weight (DCW) was determined by a precalibrated curve (1 OD_600_ = 0.375 g/liter DCW). Glucose was measured by bioanalyzer (SBA-40 C; Biology Institute of Shandong Academy of Sciences, Jinan, China). L-arginine concentration was determined by high-pressure liquid chromatography (HPLC) (Agilent Technologies, Waldbronn, Germany).

### Electronic supplementary material

Below is the link to the electronic supplementary material.

Supplementary Material 1: Primers used in this study and supplementary figures

## Data Availability

All data involved in this study are included in this published article and its additional files.
