# Supplementary material for: The flavohaemoprotein hmp maintains redox homeostasis in response to reactive oxygen and nitrogen species in Corynebacterium glutamicum
Source: Microb Cell Fact. 2023 Aug 18;22:158. doi: 10.1186/s12934-023-02160-9 (PMC10436651; doi:10.1186/s12934-023-02160-9)
Supplement: Supplementary file 1 — Supplementary Material 1: Primers used in this study and supplementary figures [file 12934_2023_2160_MOESM1_ESM.docx]

**Supplemental files**

**Table S1 Strains and plasmids used in this work.**

| Strain/plasmid | Characteristic | Source |
| --- | --- | --- |
| *E*. *coli* BL21 | Host for gene expression | Novagen |
| *E*. *coli* JM109 | Host for gene cloning | TaKaRa |
| *C. crenatum* SYPA5-5 | L-Arginine producing strain | Lab stock |
| Cc-hmp | SYPA5-5 with pXMJ19-*hmp* | This work |
| Cc-Δhmp | SYPA5-5 with deletion of *hmp* | This work |
| Cc-ΔarnR | SYPA5-5 with deletion of *arnR* | This work |
| Cc-ΔarnR-hmp | SYPA5-5 with deletion of *arnR* and pXMJ19-*hmp* | This work |
| plasmid |  |  |
| pXMJ19 | PBL1 replication, Cm^r^, P_tac_, *lacI*^q^, *C. glutamicum/E. coli* shuttle vector | Lab stock |
| pK18*mobsac*B | Kan^r^; shuttle vector for the construction of deletion mutants | Lab stock |
| pXMJ19-hmp | A derivative of pXMJ19, harboring *hmp* gene | This work |
| pK18-Δhmp | For in-frame deletion of *hmp* | This work |
| pK18-Δ*arnR* | For in-frame deletion of *arnR* | This work |

**Table S2. The primers used in construction of the recombinant strains**

| Primer name | DNA sequence (5’-3’) | Restriction sites |
| --- | --- | --- |
| pX19-hmp-F | CAGGTCGACTCTAGAGGATCCAAAGGAGGAAAATCGTGATCGTTTCCACCCAGCCCA | *Bam*H Ⅰ |
| pX19-hmp-R | CCAAAACAGCCAAGCTGAATTCTTAGGAAATCAGCCAGTCGTTTGGTGAGAACAGCTCGAAGTTC | *Eco*R Ⅰ |
| pK18-Δhmp-1 | AAACAGCTATGACATGATTACGAATTCTGTGTAGGTTTGTTCGGAG | *Eco*R Ⅰ |
| pK18-Δhmp -2 | CCCATCCACTAAACTTAAACAAAGCTTGATCACGGGGTTTTC |  |
| pK18-Δhmp -4 | TGTAAAACGACGGCCAGTGCCTCGCGATGAGCAGGGATTATTC |  |
| pK18-ΔarnR-1 | CTATGACATGATTACGAATTCGCTGTACCACTTGAGGTGCAGG | *Eco*R Ⅰ |
| pK18-ΔarnR -2 | CCCATCCACTAAACTTAAACATCACGTGTTCAGCGAGTAATTCAGC |  |
| pK18-ΔarnR -3 | TGTTTAAGTTTAGTGGATGGGTGTGGTCACGTCGGGCTC |  |
| pK18-ΔarnR -4 | ACGACGGCCAGTGCCAAGCTTACTACCGCGAAACCATCTCC | *Hin*d Ⅲ |

**Table S3. The RT-qRCR primers used in this study.**

| Primer name | DNA sequence (5’-3’) |
| --- | --- |
| 16s-RT F/R | GCCCAGGTAAGGTTCTTC/ GGTGTAGCGGTGAAATGC |
| SodA-RT F/R | CCACTCCAAGCACCACG/ GAACTTAGCGAAAGAACCGA |
| KatA-RT F/R | ACACCCCAACCTTCTTCC/ CCAAACTGGCTTACCTTCA |
| HemH-RTF/R | TGAACAAATGGCTGATGACG/ GGTGGAGACAAAACGAGGG |
| TrxA-RT F/R | GCCCCAACCTACGAGAA/ CGACCTGGCGACGAAC |
| TrxB-RT F/R | CAATCCGCCGTAGCACC/ CCACCGTATTCATAGCCCTC |
| Tpx-RT F/R | GCAACGAAACTGCTACCTC/TCAACGGATGGGAAGATG |
| WhiA-RT F/R | GCCTCAAGCGGGAAAG/ CAACGATGCCTGACGGT |
| WhiB-RT F/R | GCTGCCCAGTTACATCCC/ CGGTTTCCAGAGCCCAT |
| MsrB-RT F/R | GCCTGTGGCGAAGAGTT/ CGGTGGAGCGGAATAAC |
| MshC-RT F/R | TTCAGCAATCGCTACCAAC/ GTGAGGCGGGAAACAAA |
| Mtr-RT F/R | TTGGCGATGTCTCCTCC/ CATTGCCCAGCCGTAA |
| *Hmp*-RT F/R | TTCCATCAGGTGCGTCCGTGTA/ GGCTGCTCTTCCAACGCTTCAA |
| NarH-RT F/R | CGATGATTACTACGAGCCG/ CAAGCGTGGTGGTGGAT |
| NarJ-RT F/R | GCACCCATACTGGCAAAA/ GGAGCGGAAGGACATCAA |
| NarI-RT F/R | CGTTCCTCTGGGTTGCC/ AGTTTGGATTCGTAGATTTGG |
| GlnA-RT F/R | CGAGCATCACTGAAAACGCC/GCAACCTGGGGTGTATCTAATCG |
| GltD-RT F/R | GTTGTCATCATCGGTGGCGG/GTGCGGAAGAGGTTGGGGTA |
| GltB-RT F/R | TGGCTTCTGAATCGGGAGTGT/AGTACGCTTTACGACGCTCTCC |
| GDH-RT F/R | TGGTCATCAACCCAAGGCAC/GGAAAAGGACCCTCATTACGCT |
| AmtRTF/R | CTCGTCAAAAGTCCGTGCTCAA/GCGATTGATTGGGTTCCATAAGA |
| GlnD-RT F/R | CCAGCACTTGGGATAACAGCC/TTCTGAGCAGGGCAAAAATAGC |
| ArgC-RT F/R | GCGTTACCGCAGAACAGGCT/ACTGCTTGGGTTTGTGGCTGT |
| ArgJ-RT F/R | GACTGTCGGCGGAATGGG/ACGGTGTCATTGGTGGAGGTT |
| ArgB-RT F/R | CAAGGGTGGTTTCCGTGTGAC/CGGAGGTTCCCACAGCGTAA |
| ArgD-RT F/R | ATGCCAAGCGGTGTGGAGTT/AAGATAGCAGCCACATCCGTTG |
| ArgF-RT F/R | TCACAGATGGGTAAGGGCGAG/GAGTTCACCAGCGGCACAGT |
| ArgJ-RT F/R | GGTTGAGGACCGTCTCGTGG/CGCCTGGTGCTTCGTAGATTTC |
| ArgH-RT F/R | CAGGCAAGACCCACTTCCAG/TTGTCCAGGTCACGGATACGA |


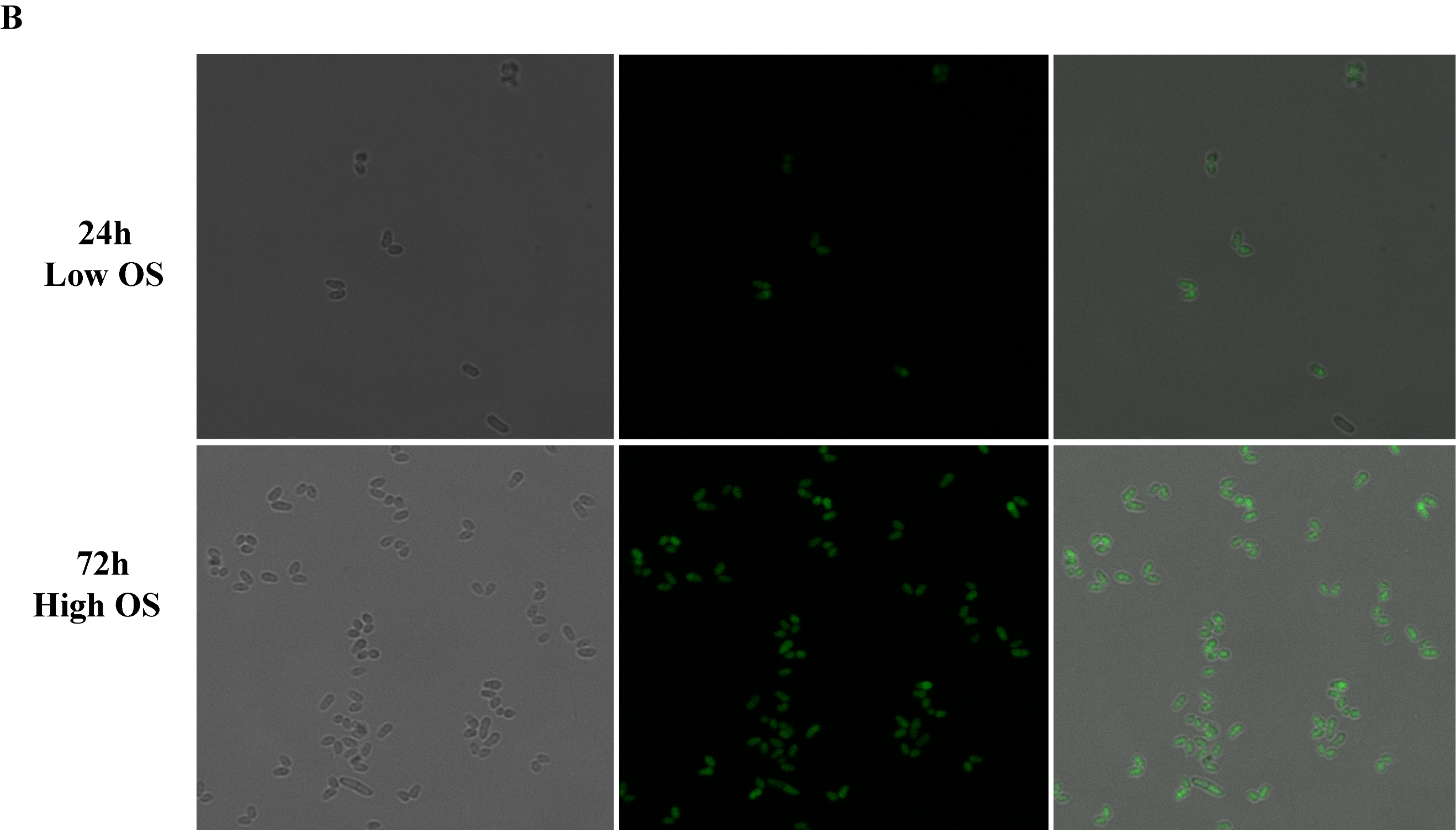

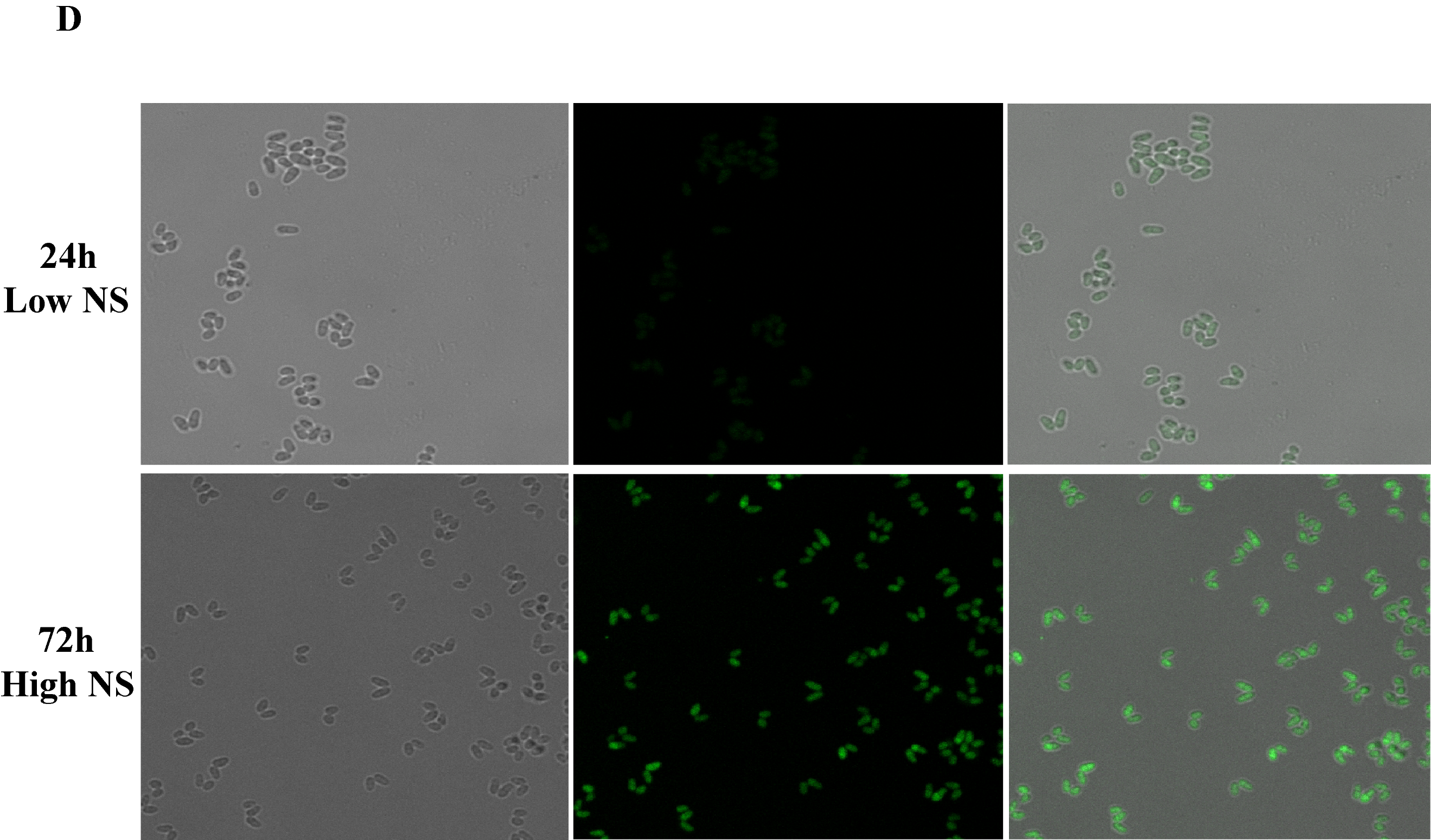


**Fig S1.** Confocal laser scanning microscope captures the intracellular ROS and RNS levels of Cc5-5 in different oxygen and nitrogen supply fermentation processes at different periods. The confocal laser scanning microscope detection conditions are excitation wavelength 502 nm, and emission wavelength 530 nm.


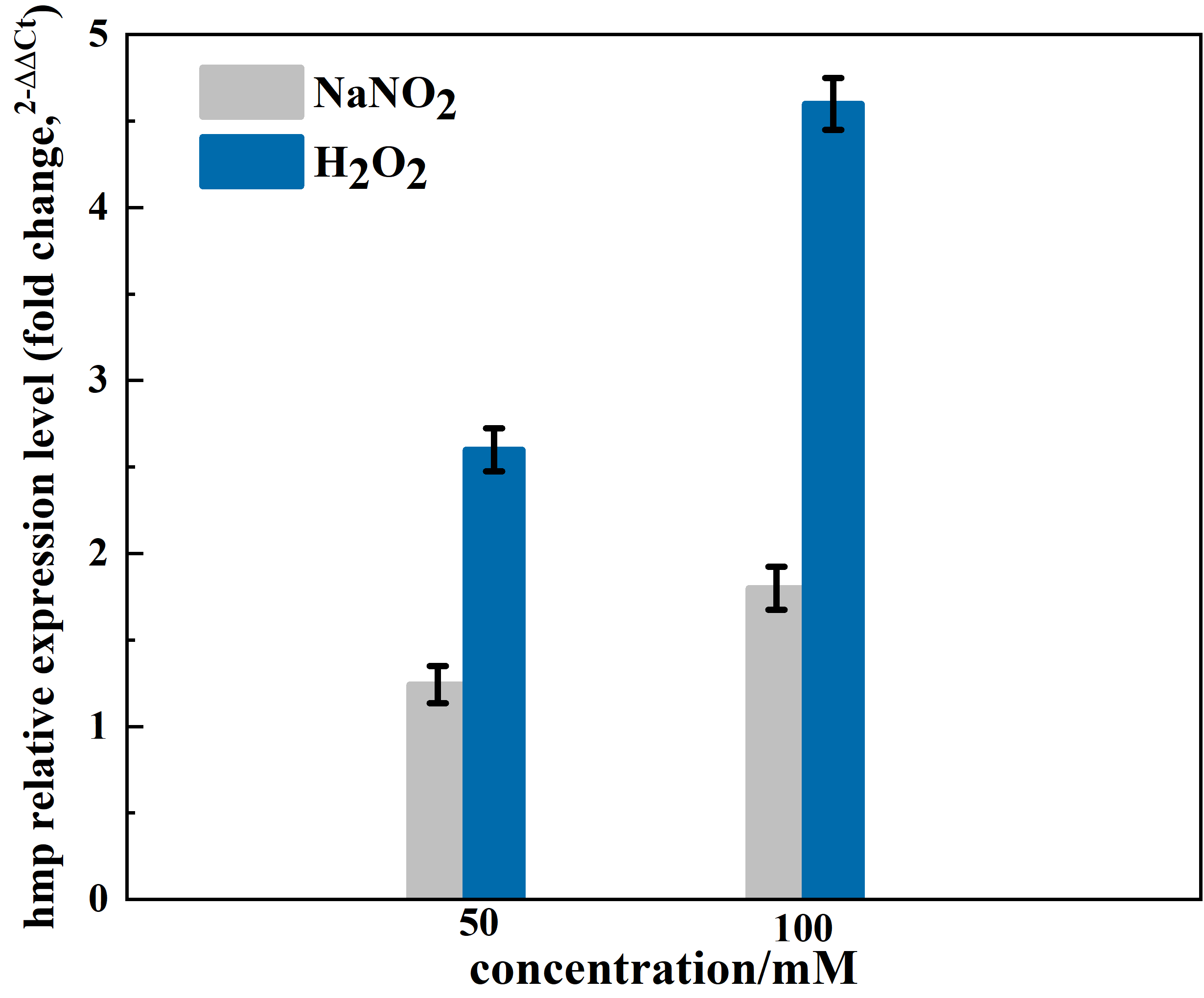


**Fig S2.** RT-qPCR analysis of *hmp* gene expression changes in Cc5-5 when exposed to different H2O2 and NaNO2 treatment concentrations.


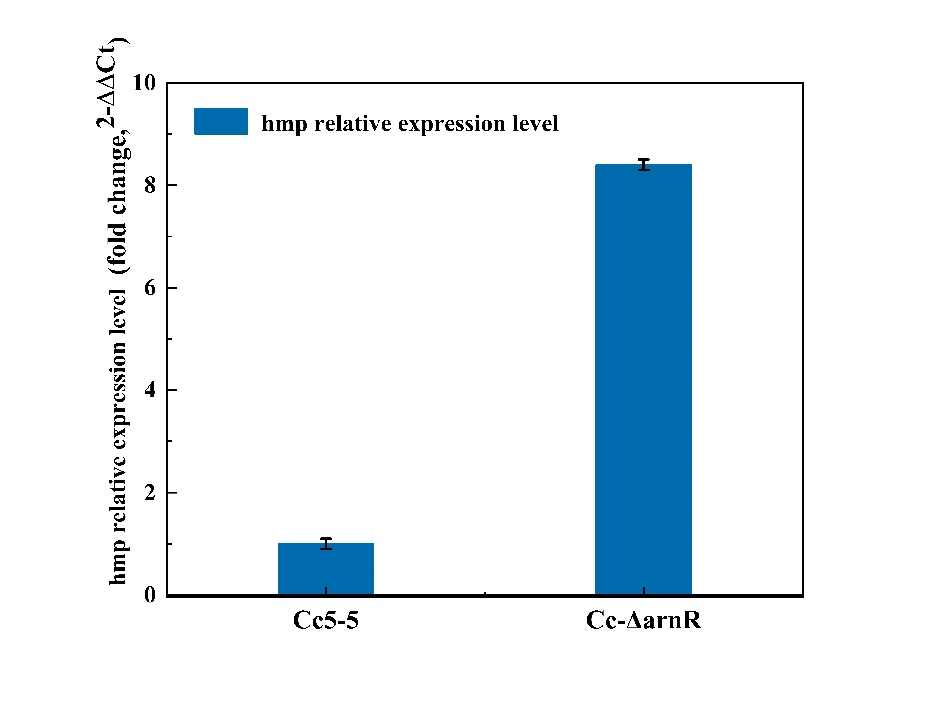


**Fig S3.** RT-qPCR verifies the regulatory effect of the transcriptional regulator ArnR on Hmp.
